# Extensive nrDNA ITS polymorphism in *Lycium*: Non-concerted evolution and the identification of pseudogenes

**DOI:** 10.3389/fpls.2022.984579

**Published:** 2022-08-25

**Authors:** Jiao Zhang, Xiulian Chi, Juying Zhong, Alisdair Fernie, Saleh Alseekh, Luqi Huang, Dan Qian

**Affiliations:** ^1^Beijing Key Laboratory of Research of Chinese Medicine on Prevention and Treatment for Major Diseases, Experimental Research Center, China Academy of Chinese Medical Sciences, Beijing, China; ^2^State Key Laboratory Breeding Base of Dao-di Herbs, National Resource Center for Chinese Materia Medica, China Academy of Chinese Medical Sciences, Beijing, China; ^3^Max Planck Institute of Molecular Plant Physiology, Potsdam, Germany

**Keywords:** *Lycium*, internal transcribed spacer (ITS), polymorphism, non-concerted evolution, pseudogenes, phylogeny

## Abstract

The internal transcribed spacer (ITS) is one of the most extensively sequenced molecular markers in plant systematics due to its generally concerted evolution. While non-concerted evolution has been found in some plant taxa, such information is missing in *Lycium*. Molecular studies of six species and two variants of the genus *Lycium* revealed high levels of intra- and inter-individual polymorphism in the ITS, indicating non-concerted evolution. All genomic DNA ITS paralogues were identified as putative pseudogenes or functional paralogues through a series of comparisons of sequence features, including length and substitution variation, GC content, secondary structure stability, and the presence of conserved motifs in the 5.8S gene, and the rate of evolution. Approximately, 60% of ITS pseudogenes could be easily detected. Based on phylogenetic analysis, all pseudogenes were highly distinct from their corresponding functional copies, tended to evolve neutrally, and clustered randomly together in the evolutionary tree. The results probably suggest that this ITS non-concerted evolution is related to the recent divergence between tandem repeats within the *Lycium* genome and hybridization between species. Our study complements those of pseudogenes in plant taxa and provides a theoretical basis for the phylogeny and genetic origin of the genus *Lycium* while having important implications for the use of ITS molecular markers for phylogenetic reconstruction.

## Introduction

Concerted evolution is a form of multigene family evolution in which the genes in a gene family or cluster are assumed to evolve in concert as a unit (Elder and Turner, 1995; Liao, 1999). The best-known example of concerted evolution is among multicopy nuclear ribosomal DNA (nrDNA) genes. In higher plants, nrDNA, a multigene family consisting of a number of highly repetitive sequences, is often found in tens of thousands of copies that are repeated in tandem within the genome and distributed on one or more pairs of chromosomes (Eickbush and Eickbush, 2007). Each repeat unit consists of three rRNAs (18S, 5.8S, and 26S), and the genes are separated from each other by internal transcribed spacers (ITS1 and ITS2) (Tian and Li, 2002; Álvarez and Wendel, 2003; Li et al., 2011). The ITS regions (ITS1, 5.8S, and ITS2), which are part of nrDNA, are among the most widely sequenced molecular markers in plant systematics and DNA barcoding studies because of their rapid concerted evolution within and between constituent subunits, fast evolution, and short length and the availability of universal primers (Álvarez and Wendel, 2003; Li et al., 2011; Schoch et al., 2012). The ITS region generally undergoes rapid concerted evolution via unequal crossing over, high-frequency gene conversion, and large deletions (Ganley and Kobayashi, 2007, 2011). Hence, intra-individual polymorphism has generally been considered an exception. However, with the development of sequencing technology, some studies have revealed intra-individual ITS polymorphisms in a range of taxa (Muir et al., 2001; Razafimandimbison et al., 2004; Wei and Wang, 2004; Harpke and Peterson, 2006, 2008a; Zheng et al., 2008; Xiao et al., 2010; Camila De Sousa et al., 2011; Zhou et al., 2013; Xu et al., 2015, 2017; Huang et al., 2016; Asanuma et al., 2019; Li et al., 2019), including non-hybrid diploids. Some ITS copies may even become pseudogenes due to functional degradation.

Pseudogenes are disabled copies of protein-coding genes, often referred to as genomic fossils (Balasubramanian et al., 2009; Sisu et al., 2014). However, in the context of phylogenetic reconstruction, pseudogenes (nrDNA or protein coding) can be defined as sequences whose nucleotide divergence pattern has, irrespective of their expression patterns, not been constrained by function (Bailey, 2003). With the intense interest of scholars in ITS pseudogenes, several methods for identifying ITS pseudogenes have been established, including methods considering 5.8S insertions and variants, lower secondary structure stability and GC content, higher methylation-related mutations throughout the ITS region, higher sequence diversity, and tree-based approaches (Bailey, 2003). On the one hand, pseudogenes are assumed to be free from selection pressure and functional constraints, allowing them to accumulate many mutations, and randomly aggregate in phylogenetic trees because of long-branch attraction (LBA), which prevents an accurate understanding of phylogenetic relationships among taxa and confounds attempts to recover correct phylogenetic species relationships (Kita and Ito, 2000; Mayol and Rosselló, 2001). On the other hand, ITS pseudogenes may be useful for phylogenetic analyses of closely related species when functional copies provide too little variation. ITS pseudogenes have been well obtained and studied in some plant taxa. In a study of *Naucleeae* (Razafimandimbison et al., 2004), only a small number of functional ITS sequences were identified, but a large number of ITS pseudogenes were found. These ITS pseudogenes have been used for phylogenetic reconstruction owing to their recent origin. Interestingly, the ITS pseudogenes found in *Corymbia* (Ochieng et al., 2007) recovered a better phylogeny than did functional ITS sequences. Furthermore, in research on *Cycas* (Xiao et al., 2010), ITS pseudogenes were found to be even more suitable than sister taxa for use as outgroups. It is undoubtedly important to identify ITS pseudogenes and evaluate their utility in phylogenetic reconstruction, but studies on ITS pseudogenes have been limited to only a few species, with considering more attention needed. Consequently, analysis of more species and adequate sampling of ITS copies will shed light on the mechanisms of concerted evolution, at the same time, may provide new genetic evidence for phylogenetic relationships and speciation.

Goji berry (also known as wolfberry) is an important and widely used source of edible and medicinal fruit in China. Recently, Goji berries, a so-called superfood, have become increasingly popular in Europe and the United States because of their health benefits. Goji berry comes from the fruits of the genus *Lycium*, a genus of both medicinal and economical importance in the Solanaceae family consisting of ~80 species worldwide, including the Mediterranean region and across Asia and Australia. It is widely cultivated in northwestern China as an economic plant. Due to the potential use of Goji berry as a specialty crop, production practices are under development in parts of southern Europe, such as Italy and Greece. There are seven species and three variants of *Lycium* in China, where wild populations are widely distributed. Compared with wild species, Goji cultivars were mainly derived from *Lycium barbarum* and are distributed in northwestern China. Several previous studies revealed that ITS sequences can be effectively used for species identification and genetic relationship studies of *Lycium* (Shi et al., 2008; Li et al., 2014). However, when amplifying the entire ITS, it was hard to obtain amplification and sequencing products (Xin et al., 2013). Subsequently, a study also found inaccessible or nested peaks in PCR product sequencing of *Lycium* and presumed the existence of ITS polymorphisms and pseudogenes in this genus (Chen et al., 2017). Although the popularity of wolfberry, *Lycium*, is attracting increasing attention, a gap remains in the systematic study of ITS sequences in the genus.

Based on our previous findings of polymorphisms in cultivated *Lycium* (Zhang et al., 2022), we performed sufficient sampling and systematically analyzed the nrDNA ITS sequences (ITS1, 5.8S, and ITS2) copied from the genus *Lycium* in this work. The objectives of this study were to reveal the level and pattern of intra- and inter-species ITS nrDNA variation, to verify the existence of nrDNA ITS pseudogenes and non-concerted evolution in *Lycium*, and to estimate the pseudogene effects on phylogenetic reconstruction. To the best of our knowledge, this is the first systematic study of incomplete ITS concerted evolution and pseudogenes in the genus *Lycium*.

## Materials and methods

### Plant material

In this study, 329 individuals of six species and two variants from *Lycium* were sampled. All samples were collected from fresh leaves in the field. The samples used for direct sequencing and genomic DNA cloning sequencing (*Lycium dasystemum, Lycium truncatum, Lycium ruthenicum, L. barbarum* var. *auranticarpum*, and *Lycium chinense*) were dried with discolored silica gel and stored in room temperature, and those for cDNA cloning sequencing (*L. barbarum, L. chinense* var. *potaninii* and *Lycium amarum*) were treated with liquid nitrogen and stored at −80°C (Supplementary Table S1).

### Nucleic acid isolation, PCR and RT–PCR, cloning, and sequencing

Genomic DNA was extracted from gel-dried leaves following the instruction of the Plant Genomic DNA Kit (TIANGEN, Beijing). RNA was extracted from fresh leaves using the RNA Easy Fast Plant Tissue Kit (TIANGEN, Beijing) and then treated with DNaseI to exclude DNA contaminants before first-strand cDNA synthesis was performed using the Fastking RT Kit (with gDNase). PCR amplification was carried out with two primer pairs, ITS1 (5'-TCCGTAGGTGAACCTGCGG3') and ITS4 (5'-TCCTCCGCTTATTGATAGC-3') (White et al., 1990), and P1 (5'-AACCTGCGGAAGGATCATTGTC-3') and P2 (5'-TGATATGCTTAAACTCAGCGGGTA-3') (Shi et al., 2008). PCR final reactions were performed in a 50 μl volume containing 20–50 ng of DNA template, 25 μl of 2 × SuperMix buffer, and each primer at 0.2 μM. The PCR program settings were as follows: 94°C for 5 min, 40 cycles of 94°C for 30 s, 56°C for 30 s, and 72°C for 45 s, and a final extension step of 72°C for 10 min. PCR and RT–PCR products were detected by electrophoresis in 1.0% agarose gel and then purified with a Thermo Scientific Gene JET Gel Extraction Kit. We cloned PCR products by using a Lethal Based Fast Cloning Kit. Eleven genomic clones per individual were obtained/sequenced, and 11 cDNA clones from *L. chinense* var. *potaninii* and *L. amarum* were sequenced with two primer pairs on an automatic DNA sequencer (ABI 3730XL, Thermo Fisher Scientific, USA) through SinoGenoMax. The PCR products were also directly sequenced.

### Data analyses

All the sequences were aligned with SeqMan (Clewley, 1995) (DNAStar, Madison, WI) and corrected manually. The boundaries of the ITS1, 5.8S, and ITS2 regions were determined based on the available ITS sequences of *Lycium* in GenBank.

To evaluate the presence of ITS pseudogenes in *Lycium*, the following analyses were carried out: (i) ITS sequence length, GC content, and NJ unrooted evolutionary trees were determined using MEGA 7.0 (Kumar et al., 2016). (ii) Three conserved patterns unique to seed plants were detected for 5.8S (motif 1: CGATGAAGACGTAGC; motif 2: GAATTGCAGAATCC; motif 3: TTTGAACGCA) (Harpke and Peterson, 2008b). (iii) Secondary structure prediction and estimation of the minimum free energy (ΔG at 37°C) were performed by using the web program Mfold (Zuker, 2003).

Genetic diversity information was evaluated based on the mean nucleotide difference number (*K*) and nucleotide diversity (π) of functional sequences and pseudogenes using DnaSP (Librado and Rozas, 2009), and a neutrality test (Fu and Li's D) was performed to further validate the putative pseudogenes and functional genes that we identified.

Tandem Repeats Finder (Benson, 1999) was used to detect repeats in ITS sequences. Differences in substitution rates can distinguish functional genes from pseudogenes (Buckler and Holtsford, 1996). The distribution and pattern of nucleotide substitutions in all sequences obtained with both primer pairs were studied using HYPERMUT (Rose and Korber, 2000) with cDNA clone CHB1-1 and putative functional copy LC17-1 as reference sequences, respectively.

## Results

The evolutionary pattern of nrDNA (including ITS) is usually concerted evolution, with few variations between copies, which provides a basis for direct sequencing of PCR products of ITS sequences. Therefore, we directly sequenced 311 individuals using the universal primers ITS1/ITS4, only 76 sequences succeeded in sequencing, and most samples failed to sequence or showed nested peaks. Although we have tried some approaches (optimizing PCR parameters, adding different concentrations of PCR synergist DMSO), we have still not been able to solve the problem in PCR directly sequenced. To improve the phenomenon, we selected 11 samples for cloning analysis (Supplementary Table S2). To get functional genes for pseudogene-aligned comparative analysis, we performed cDNA cloning and sequencing for samples that met the RNA extraction requirements (*L. chinense* var. *potaninii* and *L. amarum*).

A total of 309 ITS sequences were obtained from 329 samples of *Lycium*. After removing the repeat sequences, the total number of sequences was reduced to 268. Two pairs of PCR primers (ITS1/ITS4 and P1/P2) were used to amplify the ITS region. A total of 189 sequences were amplified with ITS1/ITS4, including 76 from direct sequencing, 82 from DNA cloning, and 31 from cDNA cloning. A total of 79 sequences were amplified with P1/P2, including 26 from DNA cloning and 53 from cDNA cloning. Five of these sequences (LC25-9-1, LC25-9-4, LC25-9-6, LC25-9-8, and CDN1-2) were excluded from the subsequent data analysis because of more than 100 bp deletion.

ITS pseudogenes were identified by nucleotide variations combined with sequence length, GC content, 5.8S conserved motifs, secondary structure stability and minimum free energy, evolutionary patterns, and phylogeny. We totally identified 154 putative pseudogene sequences out of 268 sequences, of which primer pair ITS1/ITS4 obtained 151 pseudogene sequences, while primer pair P1/P2 obtained only 3 pseudogene sequences. Meanwhile, all 76 sequences directly sequenced by PCR products were identified as putative pseudogene sequences.

### Length and repeats of the ITS region in *Lycium*

All ITS sequences in six species and two variants were obtained by performing direct sequencing, genomic DNA cloning, and cDNA cloning experiments. After amplification with the primer pair ITS1/ITS4, the length of the entire ITS region varied from 532 to 691 bp, with an alignment of 729 bp long. For the whole ITS, the 5.8S gene was relatively conserved, with amplicon lengths ranging from 150 to 169 bp. In contrast, the ITS1 and ITS2 regions displayed length variations from 168 to 260 bp and 185 to 294 bp, respectively. Among them, three samples in the ITS1 region (LBVA1-2, LBVA1-6, and LC17-1-12) were significantly shorter than the others, with a length of only 170 bp. In addition, the length of functional ITS cDNA paralogues showed similar variation and ranged from 119 to 286 bp, 149 to 172 bp, and 183 to 275 bp for ITS1, 5.8S, and ITS2, respectively. The average lengths of the ITS regions varied greatly among the species, with *L. amarum* being the only exception, showing a relatively uniform length of 540 bp.

The amplicon lengths of ITS, ITS1, 5.8S, and ITS2 ranged from 540 to 698 bp, 190 to 268 bp, 154 to 179 bp, and 250 to 272 bp, respectively, when using the primer pair P1/P2. In contrast to the amplification sequences obtained with the primer pair ITS1/ITS4, the 5.8S gene and ITS2 region were conserved in terms of length, and the length of functional ITS cDNA paralogues showed similar variants, including in *L. amarum*.

Using Tandem Repeats Finder, 14 and nine putative repeats were detected among the ITS sequences with the primer pairs ITS1/ITS4 and P1/P2, respectively. For the primer pair ITS1/ITS4, most of the repeats were located in the 5.8S region, while the *L. amarum* repeats were located in the ITS2 region. Compared to those for the primer pair ITS1/ITS4, most of the repeats detected with the primer pair P1/P2 were located in the ITS1 or ITS2 region, and repeats in *L. amarum* were located in the 5.8S region (Table 1).

**Table 1:** Tandem repeats found among ITS sequences in *Lycium*.

**Primer pairs**	**Consensus pattern**	**Taxon**	**Consensus size**	**Copy number**	**Percent matches**	**Location**
ITS1/ITS4	GAATCATT	*L.barbarum* var. *auranticarpum*	8	2.3	90	ITS1
	ATTCACTGAATTCTGCA	*L. chinense* var. *potaninii; L. chinense*	17	3	57	5.8S
	ATTCACTAAATTATCGCA	*L. ruthenicum*	18	2.4	70	5.8S
	ATTCACTGAATTCTGCA	*L. amarum*	17	3	57	ITS2
	TCTGGGGCGCCAAAATACGCTGA		23	2	72	ITS1
	AGTGAATTGCGAGAAGTA	*L. chinense; L. ruthenicum; L. dasystemum*	18	2.4	70	5.8S
	C	*L. chinense*	1	15	100	ITS1
	TGAATCAT		8	4.5	66	5.8S
	TGGTGTCGCGG		11	2.5	87	ITS2
	TCCCCCCATTTTTC		14	1.9	92	ITS1
	GTGAATCGCAGAATCA		16	2.6	62	5.8S
	GAATCATT	*L. dasystemum; L. truncatum; L. chinense; L. chinense* var. *potaninii; L.barbarum* var. *auranticarpum*	8	3.4	75	5.8S
	TGCAGAATACAGTGAAT		17	3	57	5.8S
P1/P2	TCAGCACGCGCG	*L. chinense*	12	2.2	85	ITS1
	TGCAGAATACAGTGAAT	*L. amarum*	17	3	57	5.8S
	GAATCATT	*L. barbarum*	8	3.4	75	5.8S
	TGGTGTCGCGG		11	2.5	87	ITS2
	CG	*L. chinense* var. *potaninii; L. barbarum*	2	10.5	78	ITS2
	GC		2	10.5	78	ITS1
	TATATTAAGAACT		12	2	76	ITS1
	GACGCGCATGCG		12	2.2	85	ITS2
	ATTCACTGAATTCTGCA		17	3	57	5.8S

For both primer pairs, the number of repeated sequences was significantly higher in wild samples than in cultivated samples. The percentages of repeated sequences in cultivated and wild samples were 44.4% and 46% for P1/P2 and 82.2% and 92.4% for ITS1/ITS4, respectively. In addition, approximately, 55.6% of the sequences obtained with primer pair P1/P2 contained two repeats, while those obtained with primer pair ITS1/ITS4 mostly had one repeat, approximately 52.3% (Supplementary Table S3).

### GC content of the ITS region

**Figure 1:**
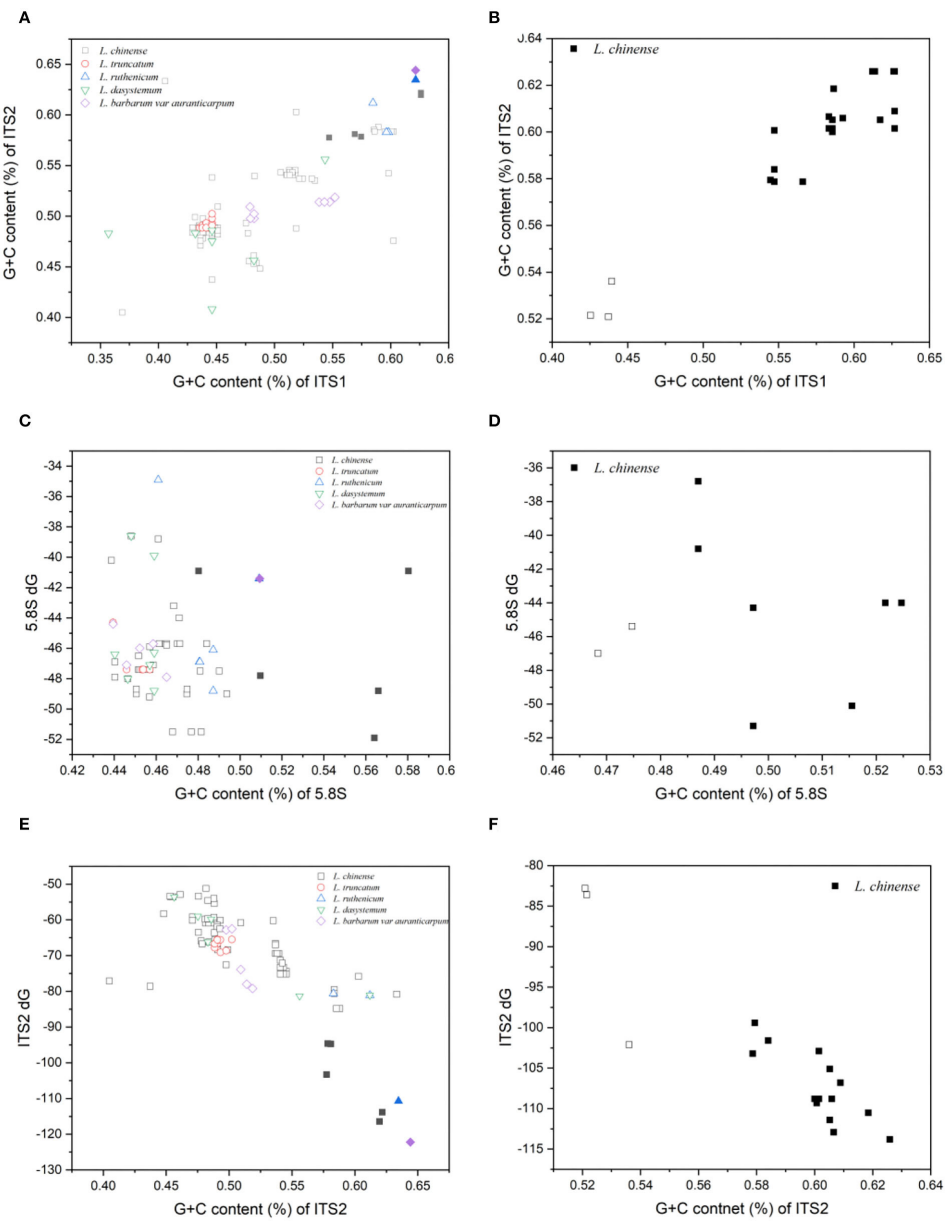
Plot of the G + C content of all paralogues obtained from five *Lycium* species. **(A,B)** ITS1 vs. ITS2. **(C,D)** 5.8S vs. the minimum free energy (Δ*G* at 37°C) of the secondary structure of 5.8S. **(E,F)** ITS2 vs. the minimum free energy (Δ*G* at 37°C) of the secondary structure of ITS2. The left panel shows the results for primer pair ITS1/ITS4, and the right panel shows those for primer pair P1/P2. Shaded symbols indicate putative functional sequences and blank symbols indicate putative pseudogenes.

Distinct clusters of clones emerged when plotting the GC contents of the ITS sequences of the two primer pairs against each other (Figures 1A,B). In most instances, the GC content correlated well between the two sets of ITS sequences. The putative functional ITS paralogues possessed high GC values in the spacers (59.8 and 59.0% in ITS1, 60.8 and 60.4% in ITS2). The GC values for 5.8S were markedly lower (53.1 and 50.9%). The average GC contents of ITS1, 5.8S, and ITS2 putative pseudogenes were 47.9% and 43.4, 46.1, and 47.1%, and 50.8% and 52.6%, respectively, approximately 2–12% lower than those of putative functional paralogues (Table 2). However, the GC contents of the functional ITS cDNA paralogues were similar to those of the putative pseudogenes, and all were lower than those of the putative functional sequences (54.2 or 54.9% for ITS1, 49.4 or 47.9% for 5.8S, and 55.8 or 56.4% for ITS2).

**Table 2:** Average lengths and GC contents of ITS regions and free energy of 5.8S secondary structures of five species of *Lycium*.

**Primer pairs**	**Code**	**No**.	**Type**	**Length(bp)(SD)**				**GC content (%) (SD)**			Δ**G(kcal**·**mol-1) (SD)**	
				**ITS**	**ITS1**	**5.8S**	**ITS2**	**ITS1**	**5.8S**	**ITS2**	**5.8S**	**ITS2**
ITS1/ITS4	LD	7	P	572 (35.4)	197.6 (18.5)	157.1 (3.0)	217.1 (31.7)	45.0 (0.0)	45.3 (0.0)	47.8 (0.0)	−45.0 (3.7)	−66.3 (9.4)
	LT^Δ^	12	P	571 (1.7)	199.3 (3.8)	153.5 (3.0)	218.2 (0.8)	44.3 (0.0)	45.1 (0.0)	49.2 (0.0)	−27.1 (0.9)	−66.8 (1.1)
	LR^Δ^	1	F	687 (0.0)	251 (0.0)	165 (0.0)	271 (0.0)	62.2 (0.0)	50.91 (0.0)	63.5 (0.0)	−41.4 (0.0)	−110.7 (0.0)
		5	P	588 (4.5)	219.8 (2.1)	155.6 (0.8)	212.6 (3.2)	59.5 (0.0)	47.9 (0.0)	58.9 (0.0)	−44.72 (5.0)	−80.7 (0.2)
	LBVA	1	F	691 (0.0)	251 (0.0)	159 (0.0)	281 (0.0)	62.2 (0.0)	50.9 (0.0)	64.4 (0.0)	−41.4 (0.0)	−122.2 (0.0)
		9	P	583.7 (24.1)	203 (23.7)	157.0 (0.0)	213.7 (0.5)	51.7 (0.0)	45.1 (0.0)	50.9 (0.0)	−46.5 (1.0)	−72.6 (7.1)
	LC^Δ^	5	F	657 (25.5)	231.2 (24.7)	154.8 (2.64)	268.2 (6.8)	58.9 (0.0)	54.0 (0.0)	59.6 (0.0)	−46.06 (4.4)	−104.56 (9.2)
		118	P	565.9 (16.4)	199.4 (13.1)	154.1 (3.7)	212.6 (11.5)	47.8 (0.1)	46.3 (0.0)	50.9 (0.0)	−47.8 (2.8)	−67.3 (8.2)
	ALL	7	F	666.1 (26.0)	236.9 (22.7)	156.9 (4.3)	270.4 (7.2)	59.8 (0.0)	53.1 (0.0)	60.8 (0.0)	−44.7 (4.3)	−108 (9.9)
		151	P	567.3 (17.0)	200.0 (14.0)	154.4 (3.6)	213.0 (12.0)	47.9 (0.1)	46.1 (0.0)	50.8 (0.0)	−47.4 (2.9)	−67.7 (7.7)
P1/P2	LC	19	F	657.5 (36.7)	229.6 (29.9)	161.3 (6.9)	266.6 (7.4)	59.0 (0.0)	50.9 (0.0)	60.4 (0.0)	−45.0 (4.1)	−108.2 (4.5)
		3	P	684.7 (0.5)	265.0 (2.2)	158.0 (0.0)	261.7 (1.9)	43.4 (0.0)	47.1 (0.0)	52.6 (0.0)	−46.5 (0.8)	−89.5 (8.9)

F, putative functional ITS sequences; P, presumed ITS pseudogene sequences; SD, standard deviation; Δ, Direct sequencing succeeded.

### Secondary structure minimum free energy of 5.8S and ITS2 rDNA

The minimum free energy (Δ*G* at 37°C) of the 5.8S and ITS2 secondary structures was predicted for all sequences. The minimum free energies for 5.8S and ITS2 of the primer pair ITS1/ITS4 were −54.6 to −34.6 kcal/mol and −122.2 to −35.5 kcal/mol, respectively. The primer pair P1/P2 yielded structures with free energies of −51.3 to −29.2 kcal/mol and −113.8 to −40.7 kcal/mol, respectively (Table 2).

Plotting the minimum free energy values of 5.8S and ITS2 vs. their GC contents resulted in similar distributions between the compared to the GC values in the spacers (Figures 1C–F). In the ITS2 region, the average value for presumed functional paralogues was significantly lower than that for the putative pseudogenes (−108 or −108.2 kcal/mol for functional copies vs. −67.7 or −89.5 kcal/mol for pseudogenes). Consistent with the GC content, the presumed functional copies showed far less variation (Table 2, Figures 1A,B).

### Nucleotide diversity and neutrality test

Sequence diversity was assessed by the average number of nucleotide differences (*K*) and nucleotide diversity (π), revealing that the sequence diversity of all putative pseudogenes was significantly higher than that of the putative functional sequences. In particular, the average number of nucleotide differences and nucleotide diversity of pseudogenes in the ITS1 region were 1.09 and 1.34 times higher, respectively, than those of functional genes, indicating that the ITS1 region accumulated more variants (Table 3).

**Table 3:** Nucleotide diversity of individual parts and the entire ITS region in *Lycium*.

**Primer pairs**	**ITS region**	**Sequence type^a^**	**Sequence number**	**Polymorphic sites**	**Total mutations**	**π^b^**	**K^c^**	**Fu and Li's D^d^**
ITS1/ITS4	ITS1	C	31	119	331	0.60701	72.234	0.96829
		F	7	169	266	0.48451	99.80952	0.57444
		P	151	168	504	0.64888	109.01168	3.57845
	5.8S	C	31	149	411	0.65802	98.045	1.35911
		F	7	140	209	0.51003	77.52381	0.07743
		P	151	150	406	0.50048	75.07223	3.42726
	ITS2	C	31	183	542	0.68977	126.228	1.73270**
		F	7	257	562	0.69472	179.23810	−0.13655
		P	151	185	552	0.62197	115.06414	3.49745
	ITS	C	31	540	1,584	0.67503	364.514	1.51015*
		F	7	591	1,000	0.58723	371.71429	0.72990
		P	151	532	1,596	0.67572	359.48342	3.63702
P1/P2	ITS1	C	52	159	450	0.59544	94.67572	1.77397**
		F	19	154	454	0.70165	108.05381	2.10623**
		P	7	126	133	0.25196	39.80952	−1.57319
	5.8S	C	52	148	386	0.64316	95.18824	1.69113**
		F	19	148	418	0.66241	98.03704	2.01404**
		P	7	157	231	0.53426	87.61905	0.17043
	ITS2	C	52	172	491	0.54802	94.259	1.34687
		F	19	169	496	0.65802	111.20539	1.94645**
		P	7	124	227	0.60789	77.80952	0.03303
	ITS	C	52	515	1,509	0.61647	317.484	1.99136**
		F	19	485	1,439	0.69542	337.27743	2.14384**
		P	7	446	959	0.68381	307.71429	−0.10613

^a^C, cDNA functional sequence; F and P as in Table 2. But primer pairs P1/P2 pseudogenes include four sequences with deletions greater than 100 bp.

^b^Nucleotide diversity (π).

^c^Average number of nucleotide differences (K).

^d^*P < 0.05, **P < 0.02.

The nucleotide diversity and an average number of nucleotide differences of pseudogene sequences differed among the three regions. In terms of nucleotide diversity, the three regions ranked as ITS2>ITS1>5.8S, while in terms of an average number of nucleotide differences, they ranked ITS1>ITS2>5.8S.

Interestingly, when using the primer pair P1/P2 to verify the results against a widely distributed species (*L. chinense*), we found that the orders of the three regions in terms of π and *K* values (ITS1>5.8S>ITS1 and 5.8S>ITS2>ITS1) were significantly different from those of the primer pair ITS1/ITS4.

Evolutionary neutrality was assessed using Fu and Li's D test on the total number of segregating sites. Although, the values of putative functional sequences obtained with ITS1/ITS4 did not show significant deviations because the presumed functional sequences accounted for only 4.6% of the pseudogene sequences. Both the functional cDNA sequences and the assumed functional sequences from primer pair P1/P2 displayed marked deviations (*P* < 0.02), indicating a deviation from neutral evolution.

### Three conserved motifs in 5.8S rDNA

In this study, motif 3 was the most conserved ITS sequence, with 91% of the sequences containing this motif. Nineteen sequences (six obtained with the ITS1/ITS4 primers and 13 with the P1/P2 primers) had at least one of the three conserved motifs of 5.8S and were therefore identified as putative functional sequences. A total of 161 sequences whose 5.8S did not contain three conserved motifs or contained at least one conserved motif with a base variation were determined to be putative pseudogenes. The base variants of the three conserved motifs of the pseudogenes showed some regularity, with the combination of T → C at position 13 of motif 1 and C → T at position 13 of motif 2 being the major variant, accounting for 83% of the overall variants.

However, only six of the 83 functional cDNA sequences contained three conserved motifs, and most of the sequences (approximately 92.8%) did not contain three conserved motifs or had base mutations, which were mainly G → A at position 12 of motif 1 and A → G at position 3 of motif 2.

### Nucleotide substitution patterns

**Figure 2:**
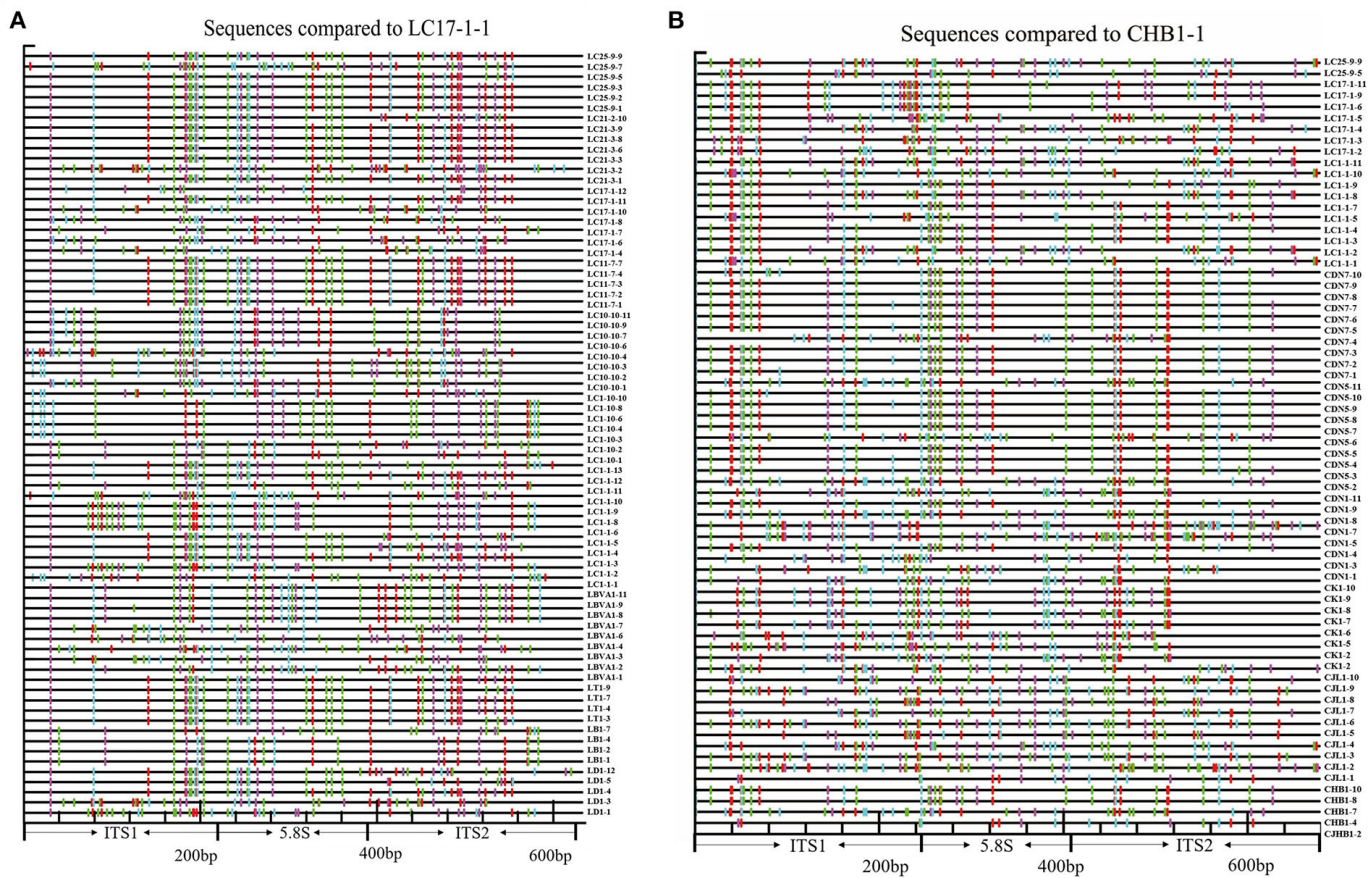
Schematic illustration of the distribution of substitution sites across the entire ITS region. **(A)** Assumed pseudogenes (excluding sequences obtained *via* direct sequencing) were yielded by primers ITS1-ITS4 using the putative functional sequence from LC17-1-1 as a reference. **(B)** Putative functional copies and cDNA paralogues from primers P1-P2 using CHB1-1 as a reference (red = GG > AG, cyan = GA > AA, green = GC > AC, magenta = GT > AT, black = not a G > A transition, yellow = gap) (For interpretation of the references to color in this figure legend, the reader is referred to the web version of this article).

The nucleotide variation profiles display the physical location of nucleotide substitutions in all putative pseudogenes from primer pair ITS1/ITS4 and all functional cDNAs and putative functional genomic paralogues from primer pair P1/P2 (Figures 2A,B).

Compared to the putative functional gene LC17-1-1, all putative pseudogenes obtained with the primer pair ITS1/ITS4 accumulated more substitutions (Figure 2A), especially the pseudogene sequences obtained by direct sequencing (Supplementary Figure S1), which were randomly distributed throughout the ITS region. In contrast, compared with the functional cDNA ITS paralogue CHB1-1, all functional cDNA and presumed functional genomic sequences obtained with P1/P2 had fewer substitutions, particularly the cDNA of the *L. barbarum* cultivar (Figure 2B). In addition, ITS sequences from the same population showed similar substitutions.

### Phylogenetic tree

**Figure 3:**
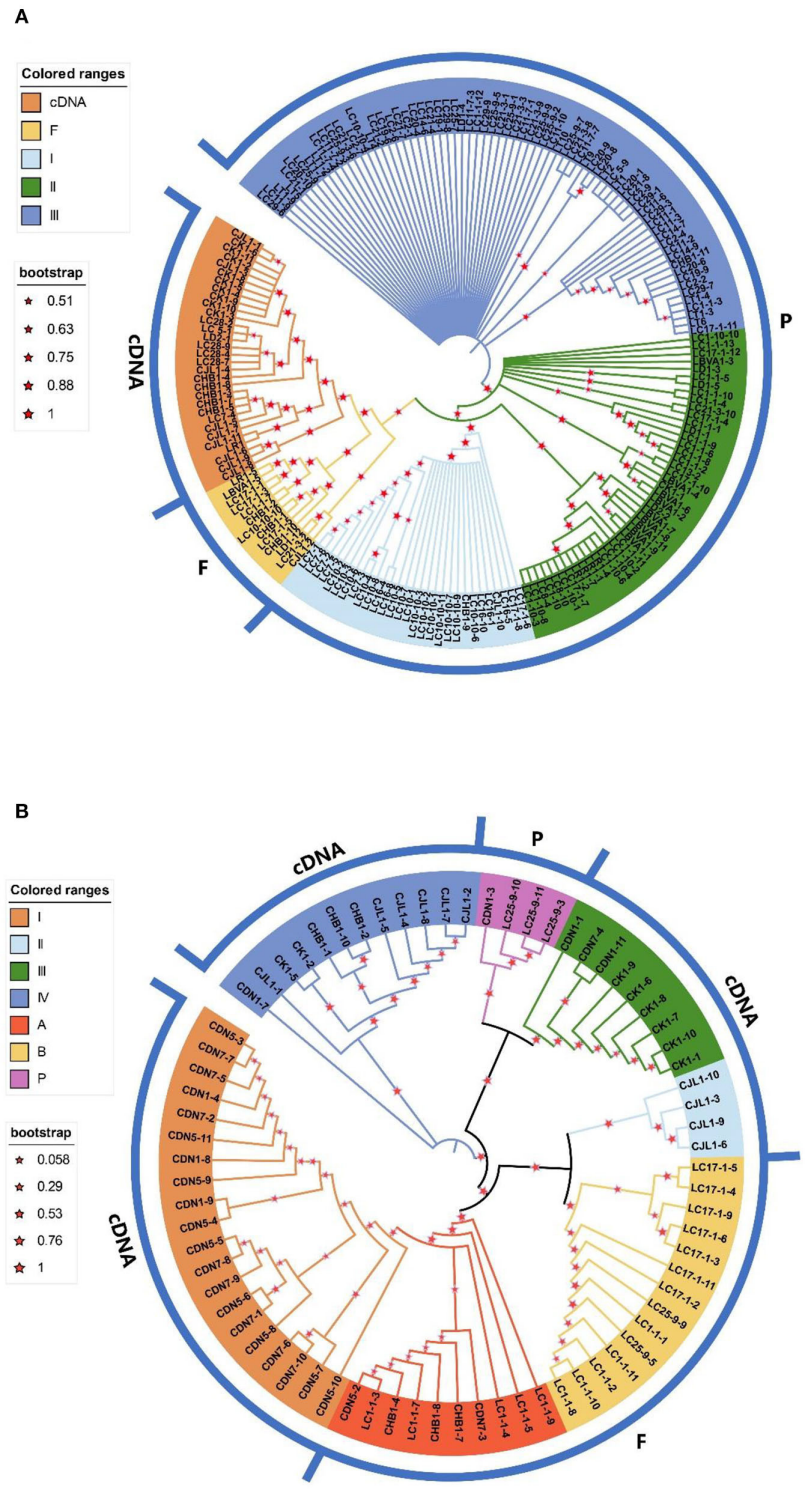
Neighbor-joining trees constructed by using the P-distance matrix from the entire ITS region of all paralogues (1,000 bootstraps). **(A)** The phylogenetic tree constructed using all sequences from the primer pair ITS1/ITS4. **(B)** The phylogenetic tree constructed using all sequences from the primer pair P1/P2. F represents presumed functional ITS sequences, P represents putative ITS pseudogene sequences, and cDNA represents ITS cDNA sequence bootstrap values >50%.

In this study, two different phylogenetic trees (A and B) were obtained by phylogenetic analysis of sequences obtained with the two primer pairs (Figures 3A,B). Tree A is the neighbor-joining (NJ) tree constructed for all sequences from primer pair ITS1/ITS4 (Figure 3A). In the NJ tree, all functional cDNA and putative functional genomic ITS copies are grouped together in a monophyletic clade with high bootstrap support (NJ bootstrap support (NJBS)=75%), with the exception of four cDNAs (CHB1-9, CHB1-11, CJL1-10, and CK1-1). The pseudogenes were found to be polyphyletic and randomly clustered into three major clades in the NJ tree. Clade I included all LC7 and LC16 sequences obtained with direct sequencing, as well as LC10 sequences obtained with direct sequencing and ITS genomic DNA copies. Clade II contained all LR, LBVA, LC1, and LC17 ITS sequences obtained with DNA sequencing, but the LC of other regions and LT and LD ITS sequences were mixed together in clade III.

Tree B is the NJ tree constructed by all ITS sequences from the primer pair P1/P2 (Figure 3B). The three putative pseudogenes (LC25-9-3, LC25-9-10, and LC25-9-11) are grouped onto one branch with high support. All functional cDNAs and presumed functional genomic ITS sequences were identified as polyphyletic and clustered onto four and two major branches in the NJ tree, respectively.

Clade I (*L. barbarum*) of functional cDNA and clade A of putative functional sequences grouped together in a monophyletic clade with high bootstrap support (NJBS=100%), and clade II (*L. chinense* var. *potaninii*) and clade B also clustered together in a monophyletic clade (NJBS=88%). The difference is that clades III and IV consisted of only functional cDNAs, while clade III consisted mainly of *L. amarum* and clade IV included *L. amarum* and *L. chinense* var. *potaninii*. The putative functional ITS copies of the two clades differed significantly, with the class B sequences being more numerous (including all genomic ITS sequences of three regions of *L. chinense*) and containing only conserved motif 2, whereas the A clade was composed of only five sequences of the same region, and all had conserved motifs 2 and 3 (Figure 3B).

The phylogenetic trees obtained for the two primer pairs were generally consistent with those obtained for resequencing data.

## Discussion

### Identification and characterization of ITS pseudogenes in *Lycium*

The highly repetitive nrDNA ITS exists in thousands of copies, and its loci are prone to loss-of-function mutations, such as insertions, deletions, or code shifts, during the evolutionary process, resulting in pseudogenes. Although there are some reports about ITS pseudogenes in some plants (Buckler and Holtsford, 1996; Muir et al., 2001; Razafimandimbison et al., 2004; Wei and Wang, 2004; Harpke and Peterson, 2006, 2008a; Zheng et al., 2008; Xiao et al., 2010; Camila De Sousa et al., 2011; Zhou et al., 2013; Xu et al., 2015, 2017; Huang et al., 2016; Asanuma et al., 2019), the severe heterozygosity of ITS sequences can lead to a search for alternative molecular markers. This is also one of the reasons why there have been fewer studies on pseudogenes. Meanwhile, the appearance of nested peaks provides opportunities for us to discover ITS polymorphisms and pseudogenes.

In this study, approximately, 60% of the 268 sequences obtained may have lost function and were identified as putative pseudogenes. Surprisingly, pseudogenes were significantly more common in the wild samples than in the cultivars for both primer pairs. Among the sequences obtained from primer pair ITS1/ITS4, 91% in the cultivar were pseudogenes, while more than 95% of the sequences in the wild samples were identified as pseudogenes. Approximately, 31% of the cultivars and 35% of the wild samples had pseudogenes when using sequences obtained with the primer pair P1/P2 (Supplementary Table S4).

Compared with functional ITS copies, putative pseudogenes had a significantly lower GC content, a lower secondary structure minimum free energy for 5.8S and ITS2, lacked conserved motifs in the 5.8S of seed plants, and increased nucleotide substitutions and sequence diversity, including the average number of nucleotide differences (*K*) and nucleotide diversity (π). The criteria that we used to identify pseudogenes were simple and effective and even showed that some of the sequences used in previous phylogenetic studies of *Lycium* were actually pseudogenes (Li et al., 2014; Shi et al., 2016).

The 5.8S gene is generally regarded as highly conserved and therefore often excluded in some studies when phylogenetic analyses are performed (Shi et al., 2016; Chen et al., 2017). It was also reported that the 5.8S gene is the most reliable indicator of functionality within the ITS region, and Yokota et al. (1989) concluded that the GC content of 5.8S functional copies in plants ranged from 50.6 to 59.3%. The average GC contents of 5.8S functional copies obtained with the two primer pairs in *Lycium* were 53.1% and 50.9%, which were similar to the values reported by Yokota et al. The average values of putative pseudogenes were 46.1 and 45.1%, which were significantly lower, indicating that frequent mutations of bases at their methylation C sites and their low GC content may lead to loss of gene function and may be the origin of pseudogenes (Bailey et al., 2003).

Evolutionary constraints can be detected by inspecting low-energy secondary structure models (Wolf et al., 2005). The minimum free energy of the secondary structure of the 5.8S and ITS2 regions in putative pseudogenes was clearly high, indicative of their lowered stability. In contrast, the secondary structure of ITS2 was more conserved in the functional sequence than that of 5.8S, as shown in Figures 1C–F. Hence, we speculated that ITS2 may be more useful for the identification of ITS pseudogenes in *Lycium*, as revealed in other taxa (Zheng et al., 2008). This may be related to the conformation of ITS2 and the spacer removal mechanism from primary transcripts (Mai and Coleman, 1997). Therefore, the low GC content and stabilized secondary structure may be reasons for the preferential pseudogene amplification in this study. In addition, all three conserved motifs specific to seed plants in the 5.8S region of the putative pseudogene were found to have base mutations. Furthermore, the pseudogenes had more significant sequence diversity. As indicated by Fu and Li's D test, the pseudogenes were not subject to selection pressure, and nucleotide substitutions are not functionally restricted during evolution and tend to evolve neutrally, allowing them to evolve faster than functional copies. Conversely, putative functional ITS paralogues were found to be evolutionarily restricted. This may be related to the maintenance of specific secondary structures during mature RNA processing (Mai and Coleman, 1997).

### ITS polymorphism and incomplete concerted evolution in *Lycium*

With the increasing development of sequencing technology, many ITS polymorphisms have been discovered among plant taxa (Muir et al., 2001; Wei and Wang, 2004; Zheng et al., 2008; Xiao et al., 2010; Xu et al., 2015). In our study, we found ITS polymorphisms in *Lycium*, as reflected not only in genomic DNA of different species and individuals but also in functional cDNA, indicating incomplete coevolution. This may be one of the reasons for the low success rate of ITS sequencing in DNA barcoding studies of *Lycium* (Ochieng et al., 2007; Chen et al., 2017). This finding also indicates the risk of using single molecular markers in plant molecular identification and improves the value of the genetic information of ITS nrDNA sequences lacking concerted evolution in the genus *Lycium*.

In this study, we found that wild samples had more extensive ITS polymorphisms than cultivars (Zhang et al., 2022). Unexpectedly, the polymorphism of the *Lycium*-specific primer pair P1/P2 was significantly lower than that of the universal primer pair ITS1/ITS4, with the putative functional sequences obtained with the latter representing no more than 10% of all sequences, while primer pair P1/P2 yielded more than 60% functional sequences (Supplementary Table S4). ITS1/ITS4 is the most popular universal primer pair for ITS fragment application, which is high universality and easy to get non-target amplicons from fungi when amplifying plant ITS fragments. This non-specific amplification phenomenon is unavoidable because fungi are symbiotic with plants in the natural ecosystems (Rodriguez et al., 2009), which may result in low PCR and sequencing success rates in this study. P1/P2 were originally designed for the identification of species in the *Lycium* genus (Shi et al., 2008). This group specificity primer pair could explain the lower pseudogene identification rate when amplifying with the P1/P2. Therefore, specific primers may be more appropriate than universal primers when performing species identification studies. Although specific primers reduced the amplification of non-targeted templates, the incomplete concerted evolution of multiple copies of ITS still exists.

In addition, we detected paralogous homologs with different ITS sequences in the same individual, including putative pseudogenes and functional paralogues. Several different pseudogenes were even detected in the same individual. For example, three different types of pseudogenes were detected in LC17-1 (Figure 3A), forming multiple lineages in the phylogenetic tree, with LC17-1-6 and LC17-1-8 clustered in clade I, LC17-1-4, LC17-1-7, LC17-1-10 and LC17-1-12 clustered in clade II, and LC17-1-11 in clade III.

The high degree of ITS polymorphism in *Lycium* was reflected not only in the GC content (40.22–57.27%) but also in the sequence length. The length ranged from 515 bp to 715 bp among all sequences, with a variation of approximately 200 bp, and intra-individual sequences were also found to differ by more than 100 bp (e.g., the shortest ITS sequence in LBVA was LBVA1-4 (540 bp), and the longest ITS sequence was 691 bp) and mainly occurred in the ITS1 region. While previous studies have shown that large ITS length variation occurs mainly among species, the presence of intra-individual variation of more than 100 bp in *Cycas* (Xiao et al., 2010) and *Lespedeza* (Xu et al., 2017) better supports our results. As in *Cycas*, no repeats were detected between ITS sequences in *Lycium* using Tandem Repeats Finder using the strictest criteria, but different short repeats (2–23 bp) were detected in all species under the most relaxed search options. These findings indicate that the presence of indels and tandem repeats plays an important role in the large amount of ITS length variation observed in *Lycium*.

The high degree of intra- and inter-individual polymorphism also suggests non-concerted ITS evolution in *Lycium*. Several biological processes might retard or disrupt concerted evolution, such as polyploidization (Karvonen and Savolainen, 1993; Suh et al., 1993), agamospermy (Campbell et al., 1997), multiple nucleolar organizer regions (NORs) (Karvonen and Savolainen, 1993; Lubaretz et al., 1996; Quijada et al., 1998; Won and Renner, 2005), longer generation times (Sang et al., 1995), and hybridization (Muir et al., 2001; Won and Renner, 2005; Grimm and Denk, 2008; Hribova et al., 2011). *Lycium* is a diploid plant (2*n* = 24 chromosomes). Therefore, genomic duplication, and therefore duplication of nrDNA loci, may not be the cause of the within-genome polymorphism observed here. The disadvantageous position of NORs (Komarova et al., 2004) on chromosomes or inactive loci without functional constraints can also lead to a slow rate of concerted evolution. Unfortunately, there is an absence of information about the number of locations of NORs in *Lycium*, but most diploid angiosperms have only one or two NORs per genome (Wendel et al., 1995). Thus, a large number of NORs may also not be the major reason for the intra-individual polymorphism and large difference in resolution patterns of the ITS clones in *Lycium*.

Hybridization is a common process that results in different copies and accounts for the high level of intra-individual polymorphism in ITS sequences found in many other plant groups, such as *Pyrus* (Zheng et al., 2008), *Cycas* (Xiao et al., 2010), *Camellia sinensis* (Xu et al., 2015), and *Lespedeza* (Xu et al., 2017). It is well known that *Lycium* is an allogamous plant, and interspecific hybridization is widespread. Therefore, it appears plausible that hybridization created a similarly high level of intra-individual ITS polymorphism in the genus. The impact of hybridization on polymorphism depends on the sequence divergence of the parents (Campbell et al., 1997); however, the number and distribution of NORs in *Lycium* are not clear. To explain the different degrees and sequence patterns of intra-individual ITS polymorphisms in *Lycium*, further related research is needed, including sequence data from other single-copy and unlinked loci, such as nuclear introns.

### Origin and phylogenetic utility of ITS pseudogenes in *Lycium*

We identified different types of pseudogenes in *Lycium*, and therefore, it is important to reveal their origin and explore their phylogenetic utility.

Compared to functional copies, pseudogenes are less functionally restricted and evolve independently at different rates, which may contribute to studies of evolutionary genetics (Ochieng et al., 2007). The ITS pseudogenes in *Lycium* were found to evolve at a high substitution rate under the standard neutral model (Table 3), and the ITS phylogeny (Figure 3) showed that functional paralogues and pseudogenes could be well separated and displayed a different phylogenetic pattern. However, due to the high number of acquired mutations, these pseudogenes clustered together randomly (Figure 3), indicating that they no longer interact genetically with functional ITS copies and could be used as another novel resource to infer phylogenetic relationships. In addition, considering the high sequence divergence between ITS pseudogenes and their corresponding functional copies, we hypothesize that the ITS pseudogenes in *Lycium* originated earlier.

Some studies have considered the phylogenetic consequences of pseudogenes. In the study of *Pyrus* (Zheng et al., 2008), it was found that functional ITS copies led to a confusing and poorly resolved phylogeny as a result of low sequence divergence, while certain types of pseudogenes and some relict pseudogenes offered more credible insight into the evolutionary history of *Pyrus* species. Similar results were obtained in *Naucleea*e (Razafimandimbison et al., 2004), clearly demonstrating that divergent putative pseudogenes are useful for phylogenetic analysis, especially when sequences of their functional counterparts are not available. Although the above two examples show that pseudogenes can be useful to some extent in resolving phylogenetic relationships in closely related species, in most cases, non-disrupted ITS evolution owing to the presence of pseudogenes poses many difficulties and even leads to misinterpretation of evolutionary relationships among taxa. In this study, divergent pseudogenes in *Lycium* accumulated large numbers of homoplastic mutations, which may be due to LBA (Anderson and Swofford, 2004), leading to random relationships among species; therefore, if pseudogenes are used together with functional sequences for phylogenetic relationship reconstruction, it will affect the stability of the system topology and preclude an accurate understanding of the phylogenetic relationships among taxa, resulting in the constructed gene trees not reflecting true evolutionary relationships. Similar results were found in *Cycas* (Xiao et al., 2010) and *Lespedeza* (Xu et al., 2017). Recombination of divergent sequences following hybridization leads to chimeric DNA sequences, which may also lead to incorrect tree inference and misestimation of evolutionary relationships. Álvarez and Wendel (2003) analyzed the recombination of divergent sequences after hybridization in several different plants and found that this process may obscure the true phylogenetic signal. Thus, when using ITS markers to reconstruct phylogenies and study the evolution process after hybridization, attention must be paid to parental homozygosity and the effects of pseudogenes.

When we validated some previous studies, we were surprised that all the putative pseudogenes and functional sequences had been clustered into monophyletic groups with high support in the evolutionary tree (Li et al., 2014; Shi et al., 2016; Chen et al., 2017) (Supplement Figure S2). In summary, although ITS pseudogenes may obscure the true phylogenetic relationships in *Lycium*, they can better explain the formation of clustering branches in phylogenetic trees reported in some previous studies.

## Conclusion

To the best of our knowledge, this is the first report of non-concerted evolution and pseudogenes of ITS sequences in *Lycium*. We revealed high polymorphism in both genomic DNA and functional cDNA ITS sequences, indicating incomplete concerted evolution. Approximately, 60% of the sequences degenerated into pseudogenes out of functional constraints and clustered randomly together in the evolutionary tree and thus may not be suitable for phylogenetic investigation. Nevertheless, the nested peaks, low identification rates, and branch formation on the evolutionary tree in previous identification studies of *Lycium* can be well explained from the perspective of ITS polymorphisms and pseudogenes. In summary, the study of ITS polymorphisms in *Lycium* not only reveals new taxa with pseudogenes but also provides a reference for germplasm resources and DNA barcoding studies of *Lycium*.

## Data availability statement

The original contributions presented in the study are included in the article/Supplementary material, further inquiries can be directed to the corresponding author/s.

## Author contributions

JZha: methodology, data curation, and writing—original draft. XC: resources and investigation. JZho: methodology and analysis. AF: writing—review and editing. SA: formal analysis and validation. LH: conceptualization, supervision, and writing—review and editing. DQ: conceptualization, funding acquisition, project administration, and writing—review and editing. All authors contributed to the article and approved the submitted version.

## Funding

This research was supported by the Fundamental Research Funds for the Central Public Welfare Research Institutes (JJPY2022008, ZZ13-YQ-079, and XTCX2021001), the National Natural Science Foundation of China (81803671), and the Scientific and technological innovation project of China Academy of Chinese Medical Sciences (CI2021A041 and CI2021B014).

## Conflict of interest

The authors declare that the research was conducted in the absence of any commercial or financial relationships that could be construed as a potential conflict of interest.

## Publisher's note

All claims expressed in this article are solely those of the authors and do not necessarily represent those of their affiliated organizations, or those of the publisher, the editors and the reviewers. Any product that may be evaluated in this article, or claim that may be made by its manufacturer, is not guaranteed or endorsed by the publisher.
