# Supplementary material for: Extensive nrDNA ITS polymorphism in Lycium: Non-concerted evolution and the identification of pseudogenes
Source: Front Plant Sci. 2022 Aug 25;13:984579. doi: 10.3389/fpls.2022.984579 (PMC9453804; doi:10.3389/fpls.2022.984579)
Supplement: Supplementary file 1 [file Data_Sheet_1.pdf]

Supplementary Matreials

Supplementary Table S1 Information on the investigated species and cultivars of *Lycium*.

Code	Common name	Type	Species	Number of population	Number of sample	Collection locality
N1	Ningqi No. 1	cultivar	*L. barbarum*	1	1	Institute of Medicinal Plant Development
N5	Ningqi No. 5	cultivar	*L. barbarum*	1	1	Institute of Medicinal Plant Development
N7	Ningqi No. 7	cultivar	*L. barbarum*	1	1	Institute of Medicinal Plant Development
JL	Beifang gouqi	cultivar	*L. chinense* var. *potaninii*	1	1	Julu, Heibei Province
HB	Beifang gouqi	cultivar	*L. chinense* var. *potaninii*	1	1	Yanqing, Beijing
LD	Xinjiang gouqi	wild	*L. dasystemum.*	2	10	Tianshui, Gansu Province
					10	Dingxi, Gansu Province
LT	Jiee gouqi	wild	*L. truncatum*	1	10	Dingxi, Gansu Province
LR	Heiguo gouqi	wild	*L. ruthenicum*	1	1	Geermu, Qinghai Province
K	Ku gouqi	wild	*L. amarum*	1	1	Tibet
LBVA	Huangguo gouqi	wild	*L. barbarum* var. *auranticarpum*	1	1	Delingha, Qinghai Province
LC1	Zhonghua gouqi	wild	*L. chinense*	29	10	Liangshan, Sichuan Province
LC2	Zhonghua gouqi	wild	*L. chinense*		10	Hengshui, Hebei Province
LC3	Zhonghua gouqi	wild	*L. chinense*		11	Enshi, Hubei Province
LC5	Zhonghua gouqi	wild	*L. chinense*		10	Xian, Shaanxi Province
LC6	Zhonghua gouqi	wild	*L. chinense*		10	Bozhou, Anhui Province
LC7	Zhonghua gouqi	wild	*L. chinense*		10	Ankang, Shaanxi Province
LC8	Zhonghua gouqi	wild	*L. chinense*		10	Dingxi, Gansu Province
LC9	Zhonghua gouqi	wild	*L. chinense*		10	Shaotong, Yunnan Province
LC10	Zhonghua gouqi	wild	*L. chinense*		10	Ganzhou, Jiangxi Province
LC11	Zhonghua gouqi	wild	*L. chinense*		10	Xiangyang, Hubei Province
LC12	Zhonghua gouqi	wild	*L. chinense*		10	Yubei, Chongqing Province
LC13	Zhonghua gouqi	wild	*L. chinense*		10	Yichang, Hubei Province
LC14	Zhonghua gouqi	wild	*L. chinense*		10	Dezhou, Shandong Province

LC15	Zhonghua gouqi	wild	*L. chinense*	11	Bozhou, Anhui Province
LC16	Zhonghua gouqi	wild	*L. chinense*	10	Liuan, Anhui Province
LC17	Zhonghua gouqi	wild	*L. chinense*	10	Xianyang, Shaanxi Province
LC18	Zhonghua gouqi	wild	*L. chinense*	10	Loudi, Hunan Province
LC19	Zhonghua gouqi	wild	*L. chinense*	10	Xiangyang, Hubei Province
LC20	Zhonghua gouqi	wild	*L. chinense*	10	Jingzhou, Hubei Province
LC21	Zhonghua gouqi	wild	*L. chinense*	8	Dezhou, Shandong Province
LC22	Zhonghua gouqi	wild	*L. chinense*	7	Luoyang, Henan Province
LC23	Zhonghua gouqi	wild	*L. chinense*	9	Chizhou, Anhui Province
LC25	Zhonghua gouqi	wild	*L. chinense*	9	Xinyang, Henan Province
LC26	Zhonghua gouqi	wild	*L. chinense*	9	Yuncheng, Shanxi Province
LC27	Zhonghua gouqi	wild	*L. chinense*	10	Sanmenxia, Henan Province
LC28	Zhonghua gouqi	wild	*L. chinense*	10	Xuzhou, Zhejiang Province
LC29	Zhonghua gouqi	wild	*L. chinense*	10	Huangshi, Hubei Province
LC31	Zhonghua gouqi	wild	*L. chinense*	7	Jiyuan, Henan Province

Supplementary Table S2 Information on two primer pairs genomic DNA clone samples.

Primer pairs	Sample	Reasons for choice
ITS1/ITS4	LC1-1	PCR product direct sequencing failure
	LC1-10	PCR product direct sequencing success
	LC25-9	PCR product direct sequencing success
	LC10-10	PCR product direct sequencing nested peaks
	LC11-7	PCR product direct sequencing nested peaks
	LC17-1	PCR product direct sequencing nested peaks
	LC21-3	PCR product direct sequencing nested peaks
	LT1	PCR product direct sequencing success
	LD2	PCR product direct sequencing failure
	LR1	PCR product direct sequencing success
	LBVA1	Adding a new variant sample
P1/P2	LC1-1	PCR product direct sequencing failure
	LC25-9	PCR product direct sequencing success
	LC17-1	PCR product direct sequencing nested peaks

Supplementary Table S3 Tandem repeats of ITS sequences in *Lycium*.

Primer pairs	Plant type	Number of sequences		Number of repeating sequences	Number of repeated sequences within an individual					
					1	2	3	4	5	6
ITS1/ITS4	wild	Direct sequencing	76	75	29	46				
		DNA cloning	82	75	31	42	2			
		cDNA cloning	31	20	17	3				
	*cultivar	Direct sequencing	10	3		2		1		
		DNA cloning	57	15	1	13	1			
		cDNA cloning	33	28	12	14	2			
P1/P2	wild	DNA cloning	37	29	21	3	2	1	1	1
		cDNA cloning	8	8	3	5				
	*cultivar	cDNA cloning	63	28	10	6	2	3	2	5

* Cultivar data include data from our previous studies

Supplementary Table S4 Average lengths and GC contents of ITS regions and free energy of 5.8S secondary structures of cultivated and wild *Lycium*.

Primer pairs	Plant Type	No.	Type	Length(bp) (SD)				GC content (%) (SD)			ΔG(kcal·mol-1) (SD)	
				ITS	ITS1	5.8S	ITS2	ITS1	5.8S	ITS2	5.8S	ITS2
ITS1/ITS4	*cultivar	54	C	570.6(36.7)	196.9(26.6)	156.6(4.2)	217.2(23.2)	50.4(0.0)	47.5(0.0)	51.7(0.1)	-47.7(3.4)	-70.3(15.9)
		6	F	690(2.8)	258.7(0.9)	160.3(0.9)	271(2.8)	57.0(0.0)	56.0(0.0)	58.3(0.0)	-52.3(0.5)	-97.8(3.1)
		61	P	641.8(35.7)	221.4(18.8)	160.1(1.7)	260.3(17.3)	55.7(0.0)	51.2(0.0)	57.3(0.0)	-49.6(2.0)	-99.5(11.7)
	wild	10	C	540.6(1.2)	125.4(16.6)	160.1(2.7)	255.1(14.0)	48.8(0.0)	50.3(0.0)	46.6(0.0)	-46.6(0.3)	-68.1(2.5)
		7	F	666.1(26.0)	236.9(22.7)	156.9(4.3)	270.4(7.2)	59.8(0.0)	53.1(0.0)	60.8(0.0)	-44.7(4.3)	-108.0(9.9)
		151	P	567.3(17.0)	200.0(14.0)	154.4(3.6)	213.0(11.9)	47.9(0.1)	46.1(0.0)	50.8(0.0)	-47.4(2.9)	-67.7(7.7)
P1/P2	*cultivar	44	C	635.4(42.7)	230.1(35.9)	155.3(8.4)	250(28.2)	55.5(0.0)	49.8(0.0)	57.2(0.1)	-44.0(5.4)	-95.2(14.8)
		13	F	681.1(3.2)	269(3.2)	154(0.0)	258(0.4)	67.2(0.0)	55.8(0.0)	69.4(0.0)	-43.1(2.4)	-124.5(1.7)
		6	P	625.5(22.5)	225.8(14.7)	154(0.0)	245.7(8.0)	57.7(0.0)	52.1(0.0)	59.9(0.0)	-43.2(0.7)	-94.1(2.5)
	wild	8	C	517.5(1.2)	173.8(9.1)	149.8(3.0)	194(12.5)	47.1(0.0)	47.3(0.0)	48.5(0.0)	-45.0(1.2)	-55.3(4.3)
		24	F	661.4(33.9)	237.0(30.6)	159.8(6.8)	264.7(7.5)	60.5(0.0)	51.5(0.0)	62.0(0.0)	-43.4(5.0)	-110.7(6.6)
		13	P	676.2(9.0)	264.5(7.1)	154.9(1.7)	256.3(5.5)	57.1(0.1)	51.3(0.0)	61.5(0.1)	-38.4(5.2)	-104.2(11.6)

C: functional cDNA ITS sequences; F: putative functional ITS sequences; P: presumed ITS pseudogene sequences; SD: standard deviation

* Cultivars contain data from our previous studies

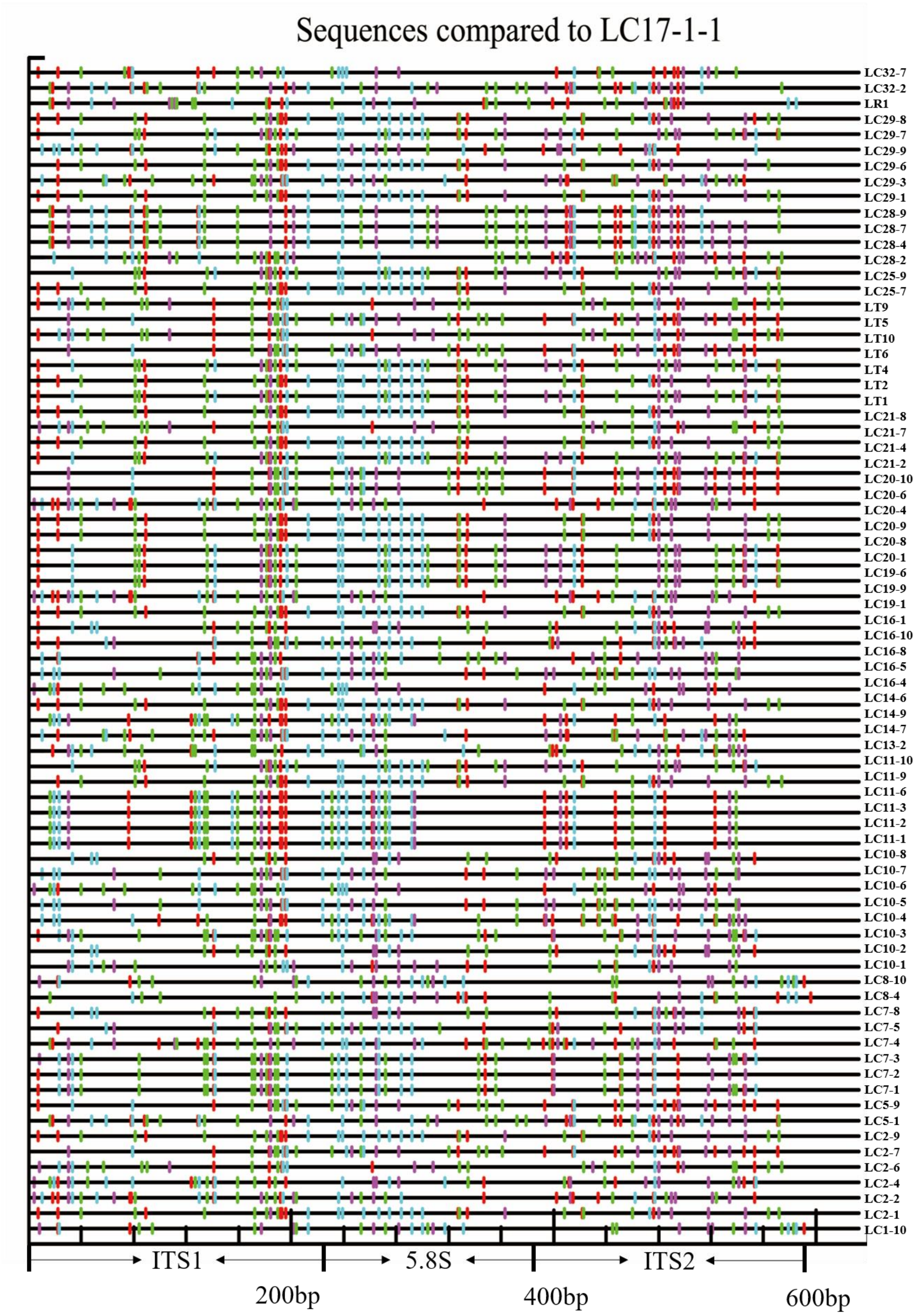

Supplementary Figure S1 Schematic of the substitution site distribution in the entire ITS region obtained by direct sequencing with the primer pair ITS1/ITS4, using the putative functional sequence of LC17-1-1 as a reference.

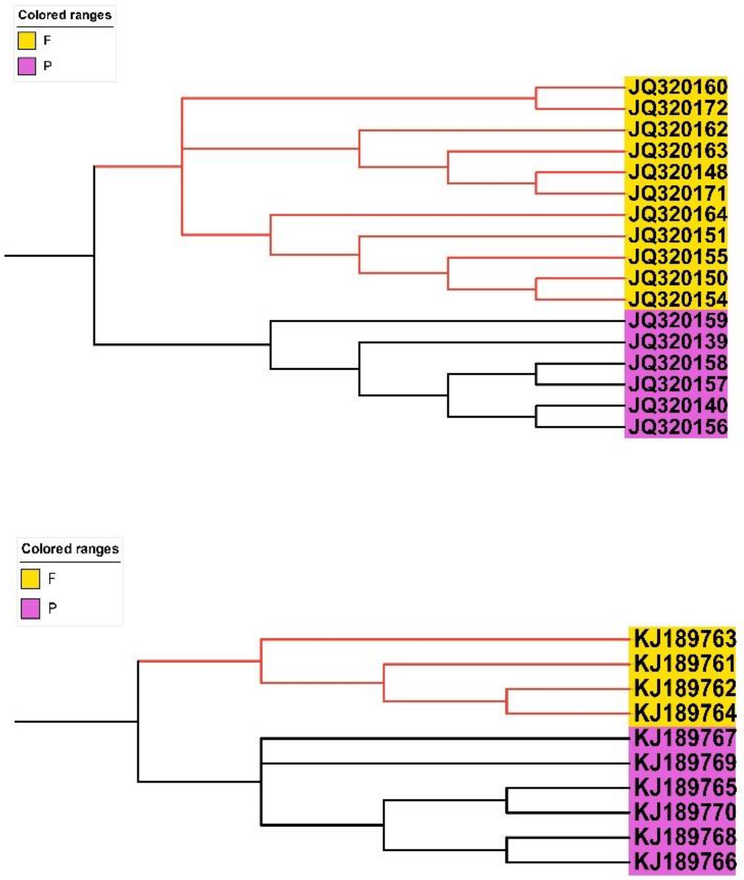

Supplementary Figure S2 Explanation of cluster formation in some studies from the perspective of pseudogenes(Li et al., 2014; Shi et al., 2016).
